# Immediate Radical Cystectomy for Massive Bleeding of Bladder Cancer

**DOI:** 10.1155/2015/154392

**Published:** 2015-12-24

**Authors:** Giovanni Cochetti, Francesco Barillaro, Andrea Boni, Ettore Mearini

**Affiliations:** Department of Surgical and Biochemical Sciences, Division of Urological, Andrological Surgery and Minimally Invasive Techniques, University of Perugia, Via Tristano di Joannuccio, 05100 Terni, Italy

## Abstract

*Objective.* To investigate feasibility and safety of our surgical strategy and clinical and oncological efficacy.* Materials and Methods.* In a high volume tertiary institution 225 radical cystectomies were performed from January 2012 to December 2014. We prospectively collected data of a cohort of 12 patients who underwent immediate open radical cystectomy for bladder cancer causing massive haematuria, acute anemia, and impossibility of postponing surgery. A retrospective study was carried out to evaluate operative data, intra- and postoperative complications, and oncologic outcomes. The Clavien-Dindo Classification was used to grade complications. The oncologic outcome was evaluated in terms of positive overall and soft tissue surgical margins and cancer specific survival at a median follow-up of 26 months.* Results.* Mean preoperative haemoglobin was 6.8 mg/dL. Mean operative time was 278 minutes. Mean blood loss was 633 mL. The overall transfusion rate was 100% with a mean of 3.6 blood units per patient before surgery and 1.8 units postoperatively. No intraoperative complications occurred. Major complications (defined as grades III, IV, and V according to Clavien-Dindo Classification) were 18,5%. In fact grade III complications were 14.8% and grade IV complications were 3.7%. Grade V did not occur. The positive surgical margin rate was 33.3% and cancer specific survival was 58,3% at median follow-up of 26 months.* Conclusions.* Immediate surgical management seems feasible, safe, and efficacious.

## 1. Introduction

Bladder cancer is the ninth most commonly diagnosed cancer worldwide, with more than 380,000 new cases each year, more than 150,000 deaths per year, and an estimated male-female ratio of 3.8 : 1.0 [1]. Radical cystectomy is the standard treatment for muscle invasive bladder cancer in most western countries [2, 3]. Although this procedure significantly compromises the patients' quality of life, it is characterized by low mortality and acceptable complication rates and is the treatment of choice for T2-4 bladder cancer at many institutions [4–6]. Salvage cystectomy is indicated in nonresponders to conservative therapy, recurrence after bladder resections, and nonurothelial carcinoma. Moreover, it is carried out as palliative treatment for fistula formation, for pain or recurrent macrohaematuria. We report for the first time, to our knowledge, a series of immediate salvage radical cystectomies for massive acute bleeding from muscle invasive bladder cancer. The aim of this study was to investigate feasibility and safety of our surgical strategy and clinical and oncological efficacy.

## 2. Materials and Methods

In a high volume tertiary institution 225 radical cystectomies were performed by a single skilled surgeon, E. M., from January 2012 to December 2014. Internal review board approved the study. We prospectively collected data from a cohort of 12 patients who underwent immediate open radical cystectomy with pelvic lymphadenectomy for muscle invasive bladder cancer causing massive haematuria, acute anemia (Hb < 8 g/dL), and impossibility of delaying the surgery; we included preoperative clinical and demographic characteristics (listed in Table 1), surgical features, and postoperative parameters. A retrospective study was carried out to evaluate operative data, intra- and postoperative complications, and oncologic outcomes with a median follow-up of 26 months (range 7–43). All the patients underwent preoperative chest and abdominal CT scan with intravenous contrast for clinical staging of bladder cancer. The Karnofsky performance status scale [7] and the American Society of Anesthesiologists (ASA) classification system were used to quantify preoperative functional status of the patients; Charlson Comorbidity Index [8] and Clavien-Dindo Classification [9] were useful in assessing the comorbidities and the surgical complications, respectively. We also evaluated surgical time, blood loss and transfusion rate, intensive care unit stay, and length of hospitalization. Follow-up visits consisted of a physical examination and serum chemistry assessment at least every 3 months for the first year, every six months for the second year, and annually thereafter. An abdominal ultrasound scan was performed 1 month after surgery in order to check the upper urinary tract status after the surgical derivation; a chest and abdominal CT scan was also repeated three and nine months after surgery for the first year, at least annually, or when clinically indicated thereafter. Tumor grade was assigned by pathologists according to the 1973 WHO grading system [10]. Pathological stage was assigned according to the 2009 American Joint Committee on Cancer TNM staging system [11]. The oncologic outcome was evaluated in terms of positive soft tissue surgical margin (STSM), positive overall surgical margins (including ureteral, urethral, and soft tissue), and cancer specific survival. We defined a positive STSM as the presence of tumor at inked areas of soft tissue on the radical cystectomy specimen. Data were analyzed with GraphPad Prism 6.0.

## 3. Results

All the patients were male, mean age was 70.6 years (ranging from 53 to 83), and mean BMI was 28.1 (range 20–35). Mean Charlson Index was 6; median Karnofsky scale was 85 (Table 1). All patients were defined as grade 4 according to ASA classification system. Due to patients' age, comorbidities were quite common. The most frequent diseases were hypertension and BPCO with a rate of occurrence of 42% (5/12). A positive history of strokes and/or aortic aneurysm was present in 33% of patients (4/12). Dyslipidemia and diabetes were common as well as with an incidence of 33% (4/12) (Table 2). All the patients showed a muscle invasive bladder cancer at CT imaging: 2/12 (17%) were cT4, 6 cT3b (50%), and 4 cT3a (33%). A clinical lymph node involvement was present in 6/12 cases (50%): 2 (17%) were cN3, 2 cN2, and 2 cN1. Metastasis occurred in 4 patients (33.3%) and all the cases were pulmonary metastasis. Mean preoperative value of haemoglobin was 6.8 mg/dL (5–7.8). Mean operative time was 278 minutes (190–500). Mean blood loss was 633 mL (500–800). The overall transfusion rate was 100% with a mean of 3.6 blood units (275 mL for unit) for patient preoperatively and 1.8 units postoperatively. In 2 cases urinary diversion was an orthotopic “Y shaped” ileal neobladder; for 4 patients an ileal conduit was performed, while in 6 patients ureterocutaneostomy was the only available option due to the comorbidities, performance status, and the age. No intraoperative complications occurred. Intensive care was required for 4/12 (33.3%) patients with a mean intensive stay of 1.25 days (1–10). Histologic exam showed high grade transitional cell carcinoma in 91% of cases (11/12) and bladder adenocarcinoma was diagnosed in one patient. Pathological staging revealed 2 cases of pT2b (17%), 4/12 (33.3%) were pT3a, and 6/12 (50%) were pT4. A nodal involvement was confirmed in 8 patients (66.6%): 4/12 were pN1, 2/12 were pN2, and 2/12 were pN3. A median of 17 lymph nodes (ranging from 12 to 21) was removed. A concomitant prostate cancer was present in 4 patients (33.3%). Overall positive surgical margins were 33.3% (4/12). STSMs were positive in 1/12 cases (8%), localized in lateral bladder wall. Clinical and pathological data are summarized in Table 3. All patients experienced at least one postoperative complication with a total of 27. According to Clavien-Dindo, grade I and II complications were 12 (44.4%) and 10 (37.1%), respectively; 4 (14.8%) grade III complications occurred: one surgical revision of the suture and two nephrostomic drainage (grade IIIa) for urinary leakage from ureteral anastomosis; one patient underwent general anesthesia for a laparotomic revision of the hemostasis (grade IIIb). Only one (3.7%) patient presented a grade IV complication due to the diagnosis of myocardial infarction at postoperative day 3. A preoperative hydronephrosis was present in 50% of patients (6/12). Three months after surgery 3/12 patients (25%) presented hydronephrosis. Mean serum haemoglobin at discharge was 9.28 g/dL (8.6–10). Mean hospital stay was 19.6 days (12–37). 8/12 (66.7%) patients were scheduled to adjuvant chemotherapy. At median follow-up of 26 months, cancer specific survival was 58.3%: 5/12 patients died from bladder cancer after a mean time of 172 postoperative days (ranging from 90 to 320 days).

## 4. Discussion

Radical cystectomy with pelvic lymph node dissection has been the gold standard therapy for muscle invasive bladder cancer for many decades, providing local cancer control and improving long-term survival [2]. However, recently, in selected patients some authors have proposed a bladder sparing combined modality therapy which includes transurethral bladder resection, external beam irradiation, and chemotherapy as an efficient alternative to radical cystectomy in order to preserve the patients' quality of life with interesting oncologic outcomes [12, 13]. Thus, salvage radical cystectomy is performed when the conservative treatment fails. Conversely, our study includes only cases which require an immediate salvage radical cystectomy as “life saving” treatment because of massive macrohaematuria. Age, overall health, and comorbidity affect the choice of primary treatment as well as the type of urinary diversion: for this reason radical cystectomy is reserved for younger patients without significant comorbidity and with a good performance status [14]. In our series mean age was 70.6 years, mean Charlson Index was 6, and median Karnofsky scale was 85. All patients were defined as grade 4 ASA especially due to acute anemia. Although each patient experienced at least one postoperative complication, major complications (defined as grades III, IV, and V according to Clavien-Dindo Classification) occurred in 18.5% of cases. This finding is comparable, but slightly higher than that of larger series [5, 6]. This is in part due to the small sample size of our study. Moreover, Hautmann et al. reported in a large single-center series early complications (within 3 months of surgery) in 58% of patients [15]. In two long-term studies and one population-based cohort study, the perioperative mortality was reported as 1.2–3% at 30 days and 2.3–5.7% at 90 days [15–17]. However, no intraoperative complication occurred in our series. Recently, many authors highlighted the matter of positive STSM and their role of predictor of oncological outcomes after radical cystectomy. In a large multicenter study Novara et al. proved that positive STSM increased the risk of disease recurrence and cancer specific mortality in patients with pT3Nany, pT4Nany, pTanyN0, and pTanyN+ disease [18]. Dotan et al. proved that positive STSM was also associated with higher rate of distant metastasis, while Hadjizacharia et al. found that it was related to worsening of overall mortality [19, 20]. The proportion of positive STSMs increases with advancing clinical T stage, advancing pathological T stage, higher pathological grade, and lymph node metastasis. Hence, the frequency of positive STSMs may depend on tumor biology (size, extension, and aggressiveness of the mass) as well as surgical factors. In our series overall positive surgical margins were 33%, but these included ureteral, urethral, and soft tissue margins. In only one patient (8%) STSMs were positive. Despite our small sample size, this finding is comparable to that of other larger series. Herr et al. proposed that positive STSM rates should be less than 10% in all corners, less than 15% for locally advanced tumors, and less than 20% for salvage RC [21]. Recently, in a 1100-patient cohort, Hautmann et al. reported after radical cystectomy a 10-year cancer specific survival of 67%, including any pT stages [22]. Culp et al. reported exciting oncologic outcomes with a 5-year cancer specific survival rate of 83.5%, but the examined cohort included only patients with cT2 muscle invasive bladder cancer without high-risk features (hydronephrosis, palpable mass, invasion into adjacent organs, and lymphovascular invasion) who were treated with radical cystectomy alone [23]. In a multicenter study of 1180 patients undergoing radical cystectomy for pT3-4 or pT0-4N1-3 Power et al. reported 2- and 5-year cancer specific survival of 67% and 53%, respectively [24]. In our series cancer specific survival was 58.3% at median follow-up of 26 months. This finding is lower than larger series but this depends on our cohort which included 17% of pT2b, 33.3% of pT3a, and 50% of pT4. Moreover, the oncological outcomes may be affected by small size of our population. If we consider the highest risk disease of our patients cohort, the oncological outcome may be considered satisfactory.

The limitations of this study are the retrospective design, the small sample size, and the short follow-up, especially for the oncological outcomes.

## 5. Conclusions

Our study has proposed for the first time, to our knowledge, radical cystectomy as immediate treatment for massive bleeding from muscle invasive bladder cancer. Although our findings cannot be considered definitive for the limitations of the study, the described surgical management seems to be feasible, safe, and efficacious.

## Figures and Tables

**Table 1:** Demographic and clinical characteristics.

Age (mean)	**70.6** *(53*–*83)*
Sex M : F	100 : 0
BMI (mean)	**28.1** *(20*–*35)*
Charlson Index (mean)	**6** *(2*–*13)*
Karnofsky scale (median)	**85** *(60*–*90)*
ASA (median)	**4**

**Table 2:** Comorbidities.

Hypertension	5/12 (42%)
BPCO	5/12 (42%)
Stroke or chronic vascular disease	4/12 (33.3%)
Abdominal aortic aneurysm	4/12 (33.3%)
Dyslipidemia	4/12 (33.3%)
Diabetes	4/12 (33.3%)
Peptic ulcer	2/12 (17%)
Concomitant tumor(s)	2/12 (17%)
Others	2/12 (17%)

**Table 3:** Clinical and pathological data.

Grade	
High	12/12 (100%)
Concomitant CIS	2/12 (16%)
cTNM	
T3a	4/12 (33%)
T3b	6/12 (50%)
T4	2/12 (16%)
N0	6/12 (50%)
N1	2/12 (16%)
N2	2/12 (16%)
N3	2/12 (16%)
M0	8/12 (67%)
M1	4/12 (33%)
pTNM	
T2b	2/12 (16%)
T3a	4/12 (33%)
T4	6/12 (50%)
N0	4/12 (33%)
N1	4/12 (33%)
N2	2/12 (16%)
N3	2/12 (16%)
M0	8/12 (67%)
Mx	4/12 (33%)
Mean lymph node removed	17 (12–21)
Urinary diversion	
UCS	4/12 (33%)
IC	4/12 (33%)
Orthotopic ileal neobladder	2/12 (16%)
Overall PSM	4/12 (33%)
Positive STSM	1/12 (8%)
Concomitant prostate cancer	4/12

UCS: ureterocutaneostomy; IC: ileal conduit; PSM: positive surgical margins; STSM: soft tissue surgical margin.
